# The Possible Role of Body Temperature in Modulating Brain and Body Sizes in Hominin Evolution

**DOI:** 10.3389/fpsyg.2021.774683

**Published:** 2022-02-10

**Authors:** Manasvi Lingam

**Affiliations:** Department of Aerospace, Physics and Space Sciences, Florida Institute of Technology, Melbourne, FL, United States

**Keywords:** neuron number, body size, hominin evolution, body temperature, foraging

## Abstract

Many models have posited that the concomitant evolution of large brains and body sizes in hominins was constrained by metabolic costs. In such studies, the impact of body temperature has arguably not been sufficiently addressed despite the well-established fact that the rates of most physiological processes are manifestly temperature-dependent. Hence, the potential role of body temperature in regulating the number of neurons and body size is investigated by means of a heuristic quantitative model. It is suggested that modest deviations in body temperature (i.e., by a couple of degrees Celsius) might allow for substantive changes in brain and body parameters. In particular, a higher body temperature may prove amenable to an increased number of neurons, a higher brain-to-body mass ratio and fewer hours expended on feeding activities, while the converse could apply when the temperature is lowered. Future studies should, therefore, endeavor to explore and incorporate the effects of body temperature in metabolic theories of hominin evolution, while also integrating other factors such as foraging efficiency, diet, and fire control in tandem.

## 1. Introduction

The human brain comprises the largest number of neurons among all primates and exhibits a high degree of efficiency, economy, and versatility (Herculano-Houzel, 2016). The datum that humans have large brains, especially in relation to their body size, is traditionally invoked to explain the emergence of their unique cognitive abilities (Jerison, 1973, 1985). It is, however, important to recognize that large brains are not necessarily better *tout court*, due to the fact that additional factors such as “modularity and interconnectivity” (Chittka and Niven, 2009) also influence several aspects of cognition (Burns et al., 2010; Avarguès-Weber and Giurfa, 2013). With this caveat in mind, the evolution of larger brains has been argued to confer a number of benefits ranging from the genesis, growth, and navigation of complex cultural societies (Byrne and Whiten, 1988; Dunbar and Shultz, 2007, 2017; Moll and Tomasello, 2007; Adolphs, 2009; Brosnan et al., 2010; Whiten and Erdal, 2012; Laland, 2017) to behavioral flexibility and general intelligence *sensu lato* (Healy and Rowe, 2006; Sol, 2008; Reader et al., 2011; Barton, 2012; Benson-Amram et al., 2016; Burkart et al., 2017).

Although humans possess the largest brains of all primates, they do not evince the largest body sizes : a list of notable primate species along with their characteristic body masses and number of neurons can be found in Table S1 of Fonseca-Azevedo and Herculano-Houzel (2012). A plethora of explanations have been propounded to account for the relatively high brain-to-body mass ratio of *Homo sapiens*. One of the most well-known class of hypotheses posits that the concurrent evolution of large brains and bodies was inhibited due to their inherent metabolic costs (Martin, 1981; Armstrong, 1983; Isler and Van Schaik, 2006). In order to overcome the metabolic costs associated with large brains—which necessitate ~20% of the total energy consumption in *H. sapiens* (Magistretti and Allaman, 2015)—a number of tenable avenues are existent in the literature.

Noteworthy examples in this category include: (1) emergence of increasingly energy-efficient foraging strategies (Bunn and Ezzo, 1993; Leonard and Robertson, 1997; Powell et al., 2017; Rosati, 2017; Thompson et al., 2019), (2) shifts in the diet, especially, toward increased carnivory (de Heinzelin et al., 1999; Milton, 1999; Braun et al., 2010; Zink and Lieberman, 2016; DeCasien et al., 2017; González-Forero and Gardner, 2018), (3) a reduction in the energy allocation to other organs (or functions) with inherently high energetic costs such as the gut (Aiello and Wheeler, 1995; Leonard et al., 2007; Antón et al., 2014) (see, however, Navarrete et al. 2011), and (4) the adoption of cooking by virtue of fire control (Wrangham et al., 1999; Wrangham, 2009, 2017; Wrangham and Carmody, 2010; Carmody et al., 2011).

Thus, for a given energy intake by way of food intake, a tradeoff between the brain and body sizes is to be expected because this energy must be partitioned between the metabolic costs of sustaining the brain and the body. Interestingly enough, some experiments tentatively support an inverse correlation between brain development and the growth in body size from birth to adulthood in humans (Kuzawa et al., 2014). By drawing upon the notion of brain-body metabolic tradeoffs, Fonseca-Azevedo and Herculano-Houzel (2012) developed a quantitative model to determine the constraints imposed on the sizes of hominin brains and bodies by metabolism. It was argued in Fonseca-Azevedo and Herculano-Houzel (2012) that the limited caloric yield of raw food substances was responsible for the relatively small brain sizes of great apes, and that a transition to cooked foods was perhaps necessary to overcome this limitation in *Homo erectus*.

As highlighted earlier, other prominent candidates for increasing the energy intake include the adoption of comparatively energy-rich diets and efficient foraging techniques. Hence, it is conceivable that one (or more) of these facets were accordingly essential for relaxing the metabolic constraints on brain and body sizes (Cornélio et al., 2016). There is, however, one crucial variable which has rarely been incorporated in current brain-body metabolic tradeoff models of hominin evolution, namely, the body temperature. This omission merits further scrutiny because there is ample empirical evidence for the sensitivity of numerous biological processes to the average body temperature, especially when it comes to the realm of metabolism (Cossins and Bowler, 1987; Brown et al., 2004; Angilletta, 2009; Clarke, 2017; Lingam and Loeb, 2021).

As a consequence, the foremost aim of this work is to illustrate how small variations in body temperature might translate to significant deviations in the brain and body sizes using a quantitative approach to draw general qualitative conclusions. To accomplish this objective, the methodology presented in Fonseca-Azevedo and Herculano-Houzel (2012) is extended to construct a simplified temperature-dependent model based on available empirical and theoretical considerations. Along the way, avenues worthy of pursuit in the future for developing more sophisticated frameworks are delineated. It is vital to appreciate at the outset that this paper merely explores the influence of temperature from a theoretical standpoint and should therefore not be regarded as being definitive in light of the numerous unknowns and uncertainties involved, but rather as analyzing different potential outcomes via a heuristic approach.

## 2. The Mathematical Framework

The details of the quantitative model are outlined, with a particular emphasis on the temperature-dependent aspects.

### 2.1. A Primer on Metabolic Scaling

The centrality of metabolism in regulating myriad biological processes is well-accepted (Blaxter, 1989; Brown et al., 2004; Schulte, 2015), although other factors (e.g., hormones, life histories, and environmental parameters) indubitably play a vital role (Glazier, 2015; Kozłowski et al., 2020). A great deal of attention has been centered on unraveling the relationship between the basal metabolic rate (BMR) and the total mass (*M*) of the organism in question (Schmidt-Nielsen, 1984; McNab, 2008; Bonner, 2011). The best-known scaling law relating these two quantities is probably Kleiber's Law (Kleiber, 1932, 1947), which states that *R*∝*M*^3/4^. The validity and universality of Kleiber's Law remains the subject of extensive debate—although ongoing studies strongly suggest that the 3/4 exponent is not universal across taxa (Dodds et al., 2001; DeLong et al., 2010; Kolokotrones et al., 2010; Glazier, 2015; Kozłowski et al., 2020), it might nevertheless be reasonably accurate for larger mammals (Clarke et al., 2010; White et al., 2019).

An oft-overlooked point, which forms the bedrock of this model, is that the BMR scales not only with the mass but also with the body temperature (*T*)—this feature is along expected lines in view of the significance of temperature in biological functions (Cossins and Bowler, 1987; Angilletta, 2009; Clarke, 2017). A widely used expression for the BMR (denoted by *R*) is


(1) $$\begin{array}{l} R\propto M^{3/4}\exp\left(-\frac{E}{k_{B}T}\right), \end{array}$$

where *k*_*B*_ is the Boltzmann constant and E signifies the activation energy for the appropriate rate-limiting step in metabolism (Gillooly et al., 2001). In other words, the above ansatz presumes that *R* is proportional to the Boltzmann–Arrhenius equation. As with Kleiber's law, the effectiveness of this function across all taxa has generated debate (Clarke et al., 2010; Knies and Kingsolver, 2010; Schulte, 2015), but this simple prescription is adopted because it constitutes a fairly robust leading-order approximation for unicellular organisms, plants and animals (Gillooly et al., 2001; Kolokotrones et al., 2010; Dell et al., 2011). Across a wide spectrum of organisms, E ranges between 0.6 and 0.7 eV in many (albeit not all) instances (Brown et al., 2004; Dell et al., 2011; Cross et al., 2015) and the mean value (0.65 eV) is nearly equal to the activation energy of 0.66 eV for ATP synthesis in mitochondria (Gillooly et al., 2006).

Let us adopt the temperature scaling in Equation (1) and consider two organisms of the same mass, although at different body temperatures of *T*_0_ and $T^{\prime }=T_{0}+\Delta T$. The ratio of the metabolic rates *R*(*T*′) and *R*(*T*_0_) (denoted by F) simplifies to


(2) $$\begin{array}{l} F\left(\Delta T\right)\approx \exp\left[\left(\frac{E}{k_{B}T_{0}}\right)\frac{\Delta T}{T_{0}}\right], \end{array}$$

after invoking the ordering Δ*T*/*T*_0_≪1, which is valid for the range of Δ*T* considered in this work. To undertake the subsequent analysis, E≈0.65 eV is utilized for reasons elucidated earlier. However, if the higher value of E≈0.94 eV for mammals espoused in Downs et al. (2008) is adopted, the results remain qualitatively similar; in quantitative terms, Δ*T* must be replaced by 1.45Δ*T* instead. The variable *T*_0_≈310 K (equivalent to *T*_0_≈37°C) serves as the “canonical” temperature for present-day *Homo sapiens*; in actuality, the average temperature is ~0.5°C lower than this value (Gurven et al., 2020; Diamond et al., 2021), but the results are not impacted to a meaningful extent. With these choices, Equation (2) can be simplified further to yield


(3) $$\begin{array}{l} F\left(\Delta T\right)\approx \exp\left(7.85\times 10^{-2}\Delta T\right). \end{array}$$

Note that Δ*T* can be either positive or negative in sign—owing to which this parameter is labeled the residual temperature—implying that F admits values both greater or smaller than unity.

### 2.2. The Quantitative Model

The question that comprises the crux of this publication is worth spelling out: What would be the consequences for brain and body sizes if hominins such as *Homo erectus* possessed an ambient body temperature of *T*′≠37°C? To answer this question, the model propounded in Fonseca-Azevedo and Herculano-Houzel (2012) is duly extended.

Three major processes are involved at the core: (a) metabolic cost of body maintenance (*E*_BD_), (b) metabolic cost of brain functioning (*E*_BR_), and (c) energetic intake via feeding (*E*_IN_). The expressions for each function are described below:


(4) $$\begin{array}{l} E_{\text{BD}}=70M_{\text{BD}}^{0.75}, \end{array}$$

(5) $$\begin{array}{l} E_{\text{BR}}=6\times 10^{-9}N, \end{array}$$

(6) $$\begin{array}{l} E_{\text{IN}}=25.4HM_{\text{BD}}^{0.53}, \end{array}$$

where the body mass *M*_BD_ is measured in units of kg, N denotes the number of neurons, and H represents the number of hours (per day) spent on feeding activities. Note that all terms on the left-hand-side are measured in units of kCal per day.

It is apparent from inspecting Equation (4) that this scaling is a consequence of Kleiber's Law. However, as outlined earlier, the metabolic rate also exhibits a thermal dependence. Hence, one must replace *E*_BD_ with a modified metabolic cost that accounts for differences in body temperature. This aspect is implemented by multiplying the right-hand-side of Equation (4) with Equation (3), as the latter embodies thermal deviations. Thus, the updated metabolic cost for body maintenance is expressible as


(7) $$\begin{array}{l} E_{\text{BD}}=70\exp\left(7.85\times 10^{-2}\Delta T\right)M_{\text{BD}}^{0.75}. \end{array}$$

When it comes to energy expenditure by primates, the major component is basal metabolism (Pontzer, 2015). The prefactor of 70 in the above equation is therefore held fixed, although variations of ≲50% are theoretically possible (Cornélio et al., 2016). As the metabolic activity of the brain is intimately connected to the rest of the body, it is reasonable to assign a similar thermal scaling for *E*_BR_ (Yu et al., 2014), which leads us to


(8) $$\begin{array}{l} E_{\text{BR}}=6\times 10^{-9}N\exp\left(7.85\times 10^{-2}\Delta T\right). \end{array}$$

Next, it is appropriate to contemplate Equation (6). The energy intake *E*_IN_ can be expressed as the product of the number of feeding hours per day (H) and the foraging efficiency (Q); note that Q would have units of energy per unit time such as kCal/hour (Cornélio et al., 2016). The allometric scaling exponent of 0.526 in Equation (6) was derived empirically (Fonseca-Azevedo and Herculano-Houzel, 2012), but a theoretical explanation is feasible. It is assumed that the foraging efficiency Q is proportional to the “search volume” (for resources) covered per unit time (V). In turn, V is modeled as being proportional to the rate of locomotion *v* and the spatial reach *w*.

Thus, if one knows the allometric scaling exponents α and β—where $v\propto M_{\text{BD}}^{\alpha }$ and $w\propto M_{\text{BD}}^{\beta }$—it is straightforward to determine $Q\propto M_{\text{BD}}^{\gamma }$ because the prior arguments indicate that γ≈α+β. As *w* should be governed by the length scale of the organism, it is reasonable to specify β = 1/3, while prior mathematical and empirical models for *v* have yielded α≈1/6-1/4 (Peters, 1983; Bejan and Marden, 2006). Therefore, the net result is γ≈0.5–0.583, which exhibits good agreement with the empirical scaling exponent of 0.526 in Equation (6).

The last component to incorporate is the thermal dependence exhibited by *E*_IN_. For starters,the Boltzmann-Arrhenius equation suggests that the energy input scales with the temperature as $\exp\left(-E_{F}/k_{B}T\right)$, where $E_{F}$ is the activation energy associated with feeding rates. By taking the ratio of the energy input rates at *T* and *T*_0_, the analog of Equation (2) is introduced, which is defined as


(9) $$\begin{array}{l} G\left(\Delta T\right)\approx \exp\left[\left(\frac{E_{F}}{k_{B}T_{0}}\right)\frac{\Delta T}{T_{0}}\right], \end{array}$$

where G(ΔT) denotes the ratio. The value $E_{F}=1.61$ eV is selected, as this was obtained for terrestrial endothermic vertebrates (Rall et al., 2012); in the absence of primate-specific data, *faute de mieux*, this estimate represents the best possible route open to us. After substituting this value of $E_{F}$ into the above expression and simplifying, it transforms into


(10) $$\begin{array}{l} G\left(\Delta T\right)\approx \exp\left(19.4\times 10^{-2}\Delta T\right). \end{array}$$

Therefore, the temperature-dependent version of *E*_IN_ is


(11) $$\begin{array}{l} E_{\text{IN}}=25.4\;H\exp\left(19.4\times 10^{-2}\Delta T\right)M_{\text{BD}}^{0.53}. \end{array}$$

An apposite criterion for facilitating the viability of a particular organism from a metabolic standpoint is *E*_IN_>*E*_BD_+*E*_BR_, where the functions are defined in Equations (7)–(11). The extremal limit is calculated by enforcing the following relation:


(12) $$\begin{array}{l} E_{\text{IN}}=E_{\text{BD}}+E_{\text{BR}}. \end{array}$$

In turn, this imposes a constraint on *M*_BD_, N, H, and Δ*T*. As the relationship between the former trio has already been investigated extensively in Fonseca-Azevedo and Herculano-Houzel (2012), the focus is primarily on Δ*T* herein.

## 3. Results

**Figure 1:**
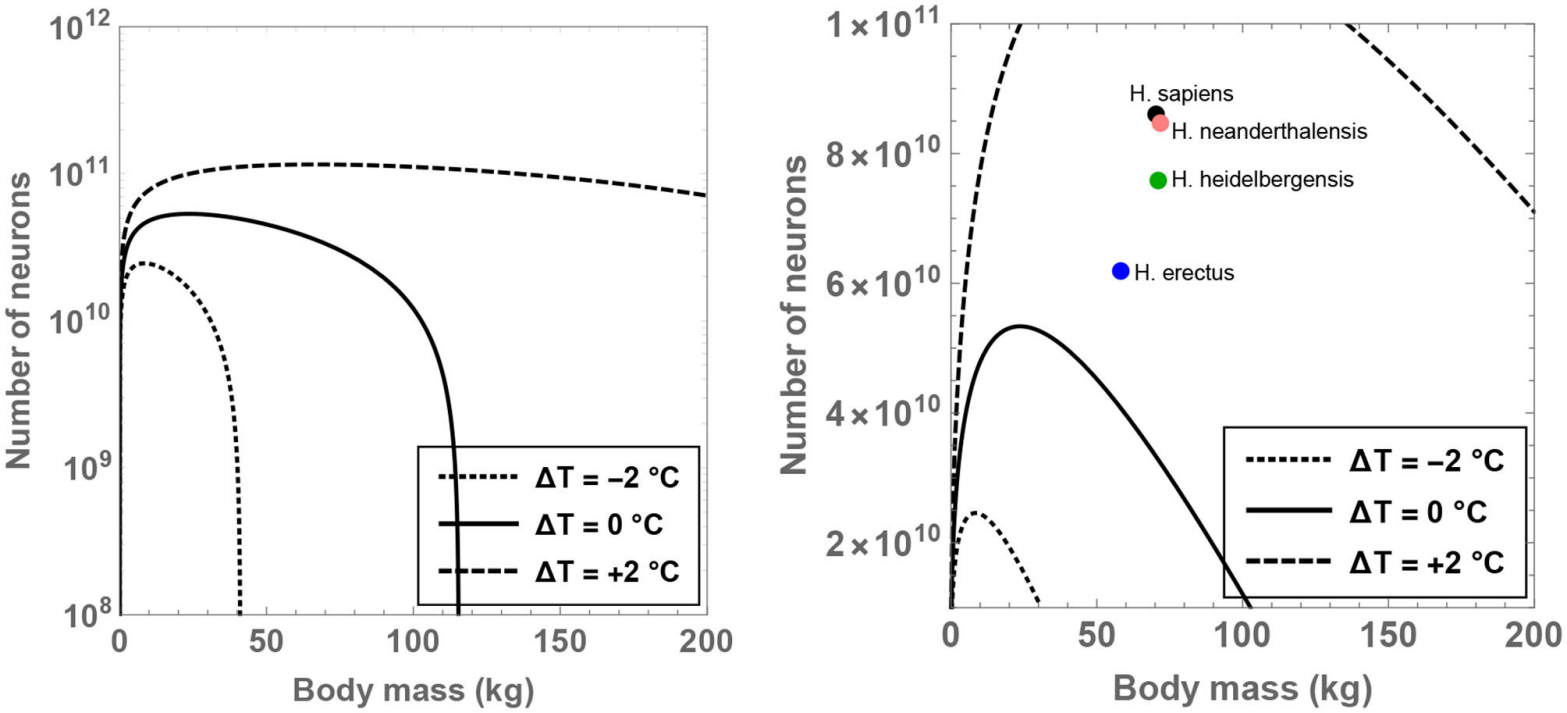
Compatible combinations of the number of neurons (N) and body mass (*M*_BD_) are shown for putative primates that engage in 8 h of feeding per day; the regions lying beneath the curves represent the permitted values. The dotted, unbroken, and dashed curves correspond to body temperatures that differ from present-day humans by −2, 0, and +2°C; the equivalent body temperatures are 35, 37, and 39°C, respectively. In the right panel (which depicts a narrower range than the left panel), the black, orange, green, and blue dots show the typical values of N and *M*_BD_ for *H. sapiens, H. neanderthalensis, H. heidelbergensis*, and *H. erectus*, respectively.

The theoretical analysis is initiated by fixing H=8 for the time being since it may constitute a sustainable upper bound on the number of hours devoted to feeding per day (Lehmann et al., 2008; Fonseca-Azevedo and Herculano-Houzel, 2012). For this choice of H, one can determine the compatible range of values admitted by *M*_BD_, N, and Δ*T*. The results have been plotted in Figure 1 for different choices of the residual temperature. It is observed that there is a significant expansion in the number of permitted neurons (for a given body mass) if Δ*T* >0 is adopted, and the converse trend is observed when Δ*T* < 0.

In particular, the right panel of Figure 1 reveals that the instantiation of certain well-known hominins that evolved after *H. erectus* is not readily feasible if these species were characterized by a body temperature equal to (or lower than) present-day humans. On the other hand, in the scenario wherein these species possessed a body temperature higher than humans by 2°C, their existence might be rendered mathematically viable. Although the data for *M*_BD_ and N for various hominins is taken from Table S1 of Fonseca-Azevedo and Herculano-Houzel (2012), the results remain essentially unchanged even if comparatively recent estimates for hominins are utilized (Grabowski et al., 2015).

In fact, specifying H=8 and Δ*T* = 1°C in conjunction with the predicted average mass of *H. erectus* (*M*_BD_ = 58 kg) yields $N\approx 7.6\times 10^{10}$, which is comfortably higher than the potential number of neurons characteristic of *H. erectus* ($N\approx 6.2\times 10^{10}$) as per some estimates (Fonseca-Azevedo and Herculano-Houzel, 2012). Likewise, after choosing *M*_BD_ = 70 kg (motivated by *H. sapiens*), Δ*T* = 1.3°C and H=8, one arrives at $N\approx 8.5\times 10^{10}$—this value happens to be close to the typical number of neurons in modern humans ($N_{\text{sapiens}}\approx 8.6\times 10^{10}$). In contrast, upon inputting Δ*T* = 0 and holding all other parameters fixed, $N\approx 3.4\times 10^{10}$ is obtained, which is evidently conspicuously lower than the requisite $N_{\text{sapiens}}$.

Next, it is instructive to tackle the crucial metric of the brain-to-body mass ratio (denoted by δ_M_) and investigate the conditions necessary for fulfilling δ_M_ = 2%. As before, the analysis is restricted to H=8 for reasons explained in the prior paragraph. To solve for δ_M_, the following relationship between δ_M_ and N from Gabi et al. (2010) is invoked:


(13) $$\begin{array}{l} N=7.0\times 10^{8}+5.7\times 10^{10}\times \delta_{\text{M}}\times M_{\text{BD}}. \end{array}$$

**Figure 2:**
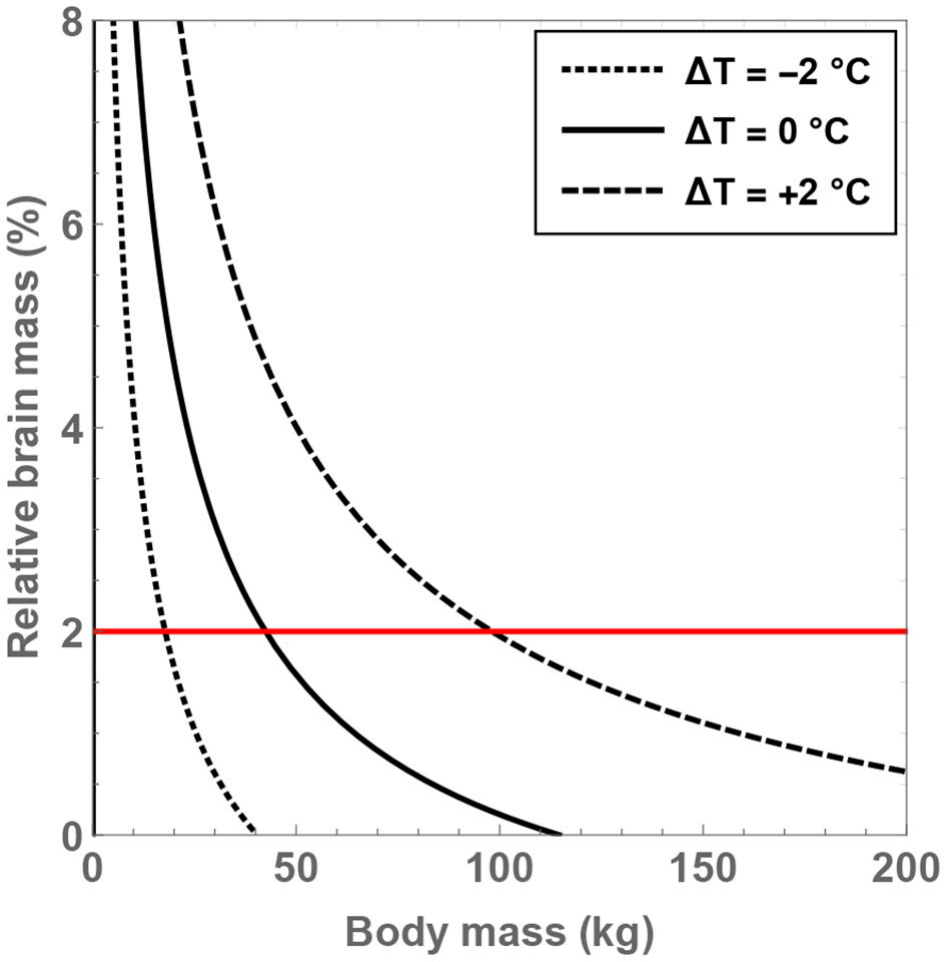
Compatible values of the brain-to-body mass ratio (δ_M_) and body mass (*M*_BD_) are depicted for putative hominins that take part in 8 h of feeding per day; the regions within the curves are the permitted domains. The dotted, unbroken, and dashed curves correspond to body temperatures that differ from present-day humans by −2, 0, and +2°C; the equivalent body temperatures are 35, 37, and 39°C, respectively. The horizontal red line is associated with δ_M_ = 2%.

One can therefore obtain δ_M_ as a function of Δ*T* and *M*_BD_ for the given value of H. Upon inspecting Figure 2 after doing so, the significance of the residual temperature in governing δ_M_ becomes manifest. For hominins with body temperatures equal to present-day humans, only modest masses of *M*_BD_ <43 kg are permitted in order to ensure that δ_M_>2%. In contrast, if body temperature is increased by 2°C, it is found that the maximal body mass that enables δ_M_ = 2% to hold true is *M*_BD_≈98 kg. As all documented hominins are less heavy than this threshold value, it may have been feasible (from a mathematical standpoint) for these species to attain a high brain-to-body mass ratio provided that they had higher body temperatures. In fact, for H=8 and Δ*T* = +1.5°C, the maximum body mass compatible with δ_M_ = 2% is estimated to be 80 kg, which is also larger than the typical masses of virtually all hominins.

**Figure 3:**
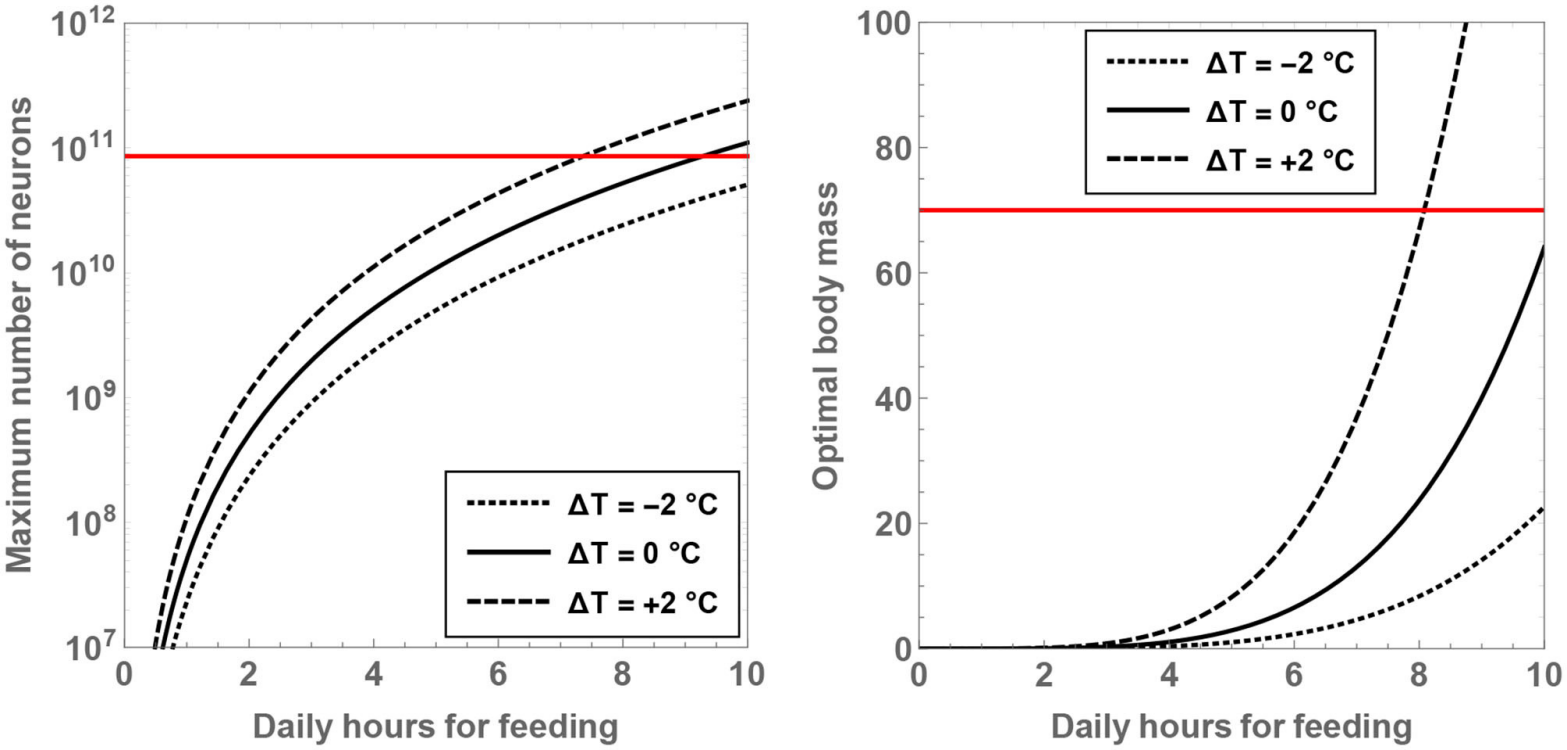
The maximal number of neurons **(Left)** and the associated body mass **(Right)** are shown as a function of the number of hours per day spent on feeding. The dotted, unbroken, and dashed curves correspond to body temperatures that differ from present-day humans by −2, 0, and +2°C; the equivalent body temperatures are 35, 37, and 39°C, respectively. The horizontal red line depicts the neuron number **(Left)** and mass **(Right)** for *H. sapiens*.

Another result worth highlighting in this context concerns the maximum number of neurons for a given value of H and Δ*T*. By solving for $\partial N/\partial M_{\text{BD}}=0$, one ends up with


(14) $$\begin{array}{l} M_{\text{max}}\approx 2.2\times 10^{-3}H^{4.5}\exp\left(0.52\Delta T\right),\\ N_{\text{max}}\approx 5.1\times 10^{7}H^{3.3}\exp\left(0.39\Delta T\right), \end{array}$$

where $N_{\text{max}}$ signifies the maximum number of neurons feasible and *M*_max_ represents the body mass of the hominin when this neuron number is attained. *M*_max_ and $N_{\text{max}}$ are plotted as a function of H and Δ*T* in Figure 3. It is noteworthy that both of these functions are strongly dependent on the two variables. More specifically, it is observed that *M*_max_ is close to the characteristic mass of *H. sapiens* when H≈8 and Δ*T* = +2°C; the corresponding value of the maximal neuron number is $N_{\text{max}}\approx 1.3\times 10^{11}$, which is higher than $N_{\text{sapiens}}$. In contrast, if Δ*T* ≤ 0, H>9 is apparently imperative in order for $N_{\text{max}}$ to overtake the characteristic number of neurons present in *H. sapiens*.

**Figure 4:**
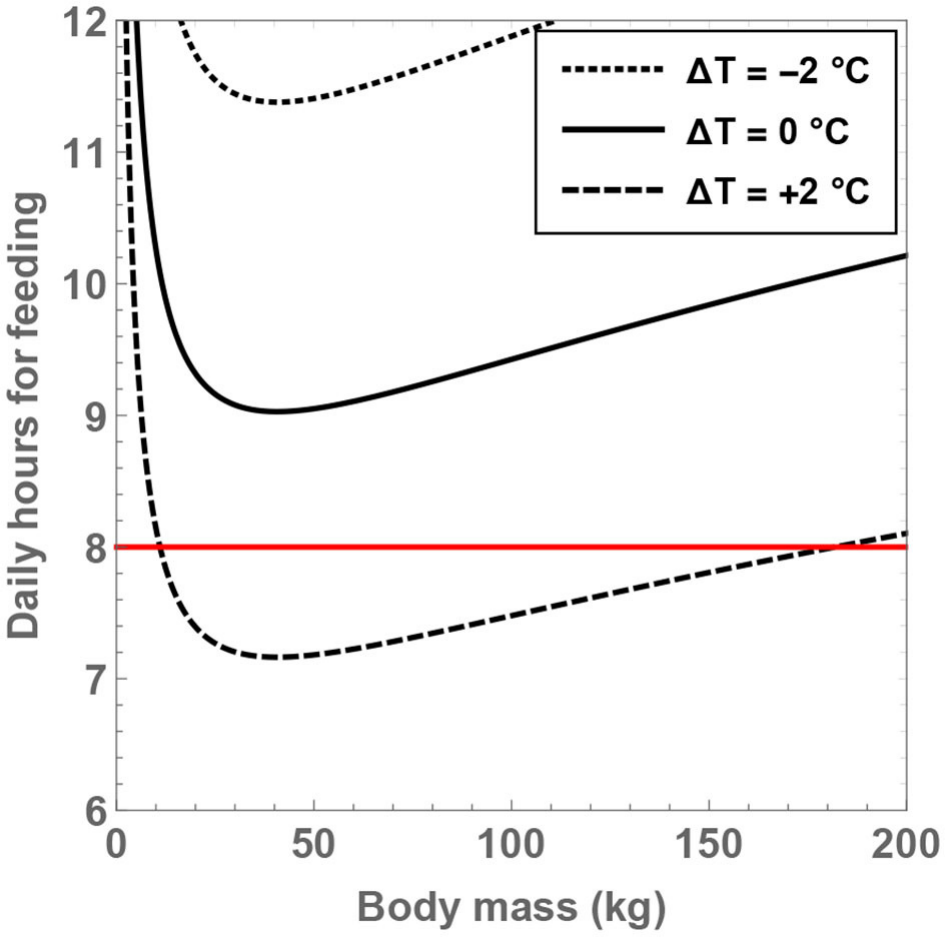
Compatible values of the number of hours per day expended on feeding (H) and body mass (*M*_BD_) are determined for putative hominins endowed with 80 billion neurons; the regions lying above the curves depict the allowed values. The dotted, unbroken and dashed curves correspond to body temperatures that differ from present-day humans by −2, 0, and +2°C; the equivalent body temperatures are 35, 37, and 39°C, respectively. The horizontal red line represents H=8 and can be regarded as a “sustainable” upper bound on H.

Hitherto, the focus was oriented toward holding the number of feeding hours fixed and determining the constraints on the other variables. It is instructive to reverse the situation and hold N fixed, thereby allowing us to gauge the permitted range of H, *M*_BD_, and Δ*T*. As the number of neurons presumably increased “only” by a factor of ~1.5 from *H. erectus* to *H. sapiens*, a fiducial value of $N=8\times 10^{10}$ can be selected without much loss of generality; this number is close to the estimated number of neurons for *H. sapiens, H. neanderthalensis*, and *H. heidelbergensis* (Herculano-Houzel, 2016). For this choice, the compatible values for the remaining variables have been plotted in Figure 4. In common with the previous figures, the results are sensitive to the temperature. In particular, it would seem impossible to achieve H≤8 when the hominins in question have a temperature equal to (or lower than) present-day humans. In contrast, when body temperature is elevated by 2°C, it is inferred that H<8 is achievable for 11 < *M*_BD_ <182 kg.

By holding N and Δ*T* fixed, it is feasible to determine the minimum number of hours that need to be expended on feeding, as well as the associated body mass, which are denoted by $H_{\text{min}}$ and *M*_min_, respectively. Thus, by solving for $\partial H/\partial M_{\text{BD}}=0$, one arrives at


(15) $$\begin{array}{l} M_{\text{min}}\approx 1.2\times 10^{-13}N^{4/3},\\ H_{\text{min}}\approx 5.0\times 10^{-3}N^{0.3}\exp\left(-11.6\times 10^{-2}\Delta T\right). \end{array}$$

One of the striking aspects of this expression is that *M*_min_ does not exhibit any dependence on the temperature, although it does scale with the number of neurons. If the value of N for *H. sapiens* is substituted, it is found that the corresponding optimal body mass is 45 kg. Although this is approximately a factor of 1.5 removed from the typical mass of *H. sapiens*, it is important to appreciate that evolution is not necessarily guaranteed to converge toward strict optimality.

**Figure 5:**
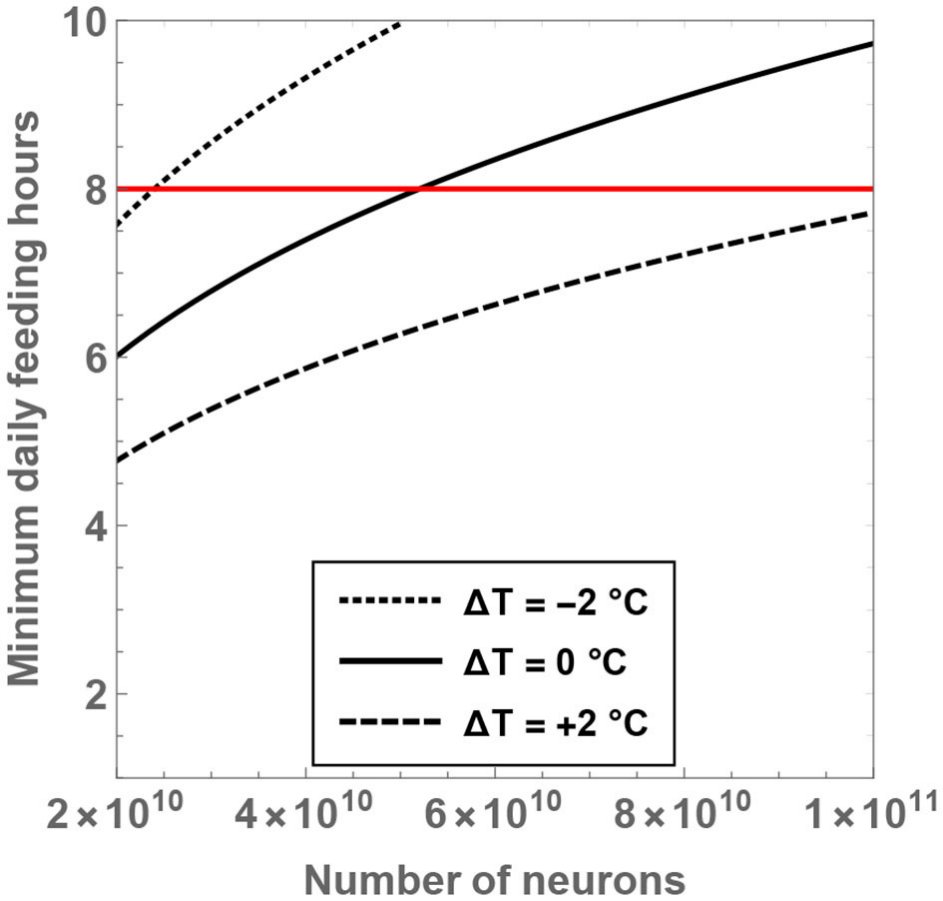
The minimum number of daily feeding hours is shown as a function of the total number of neurons. The dotted, unbroken and dashed curves correspond to body temperatures that differ from present-day humans by −2, 0, and +2°C; the equivalent body temperatures are 35, 37, and 39°C, respectively. The horizontal red line depicts the “sustainable” upper bound on the feeding time per day.

From the expression for $H_{\text{min}}$, it is seen that the minimal number of feeding hours decreases with an increase of the residual temperature, which is consistent with our prior analysis. In Figure 5, the behavior of $H_{\text{min}}$ is shown as a function of N and Δ*T*. If the temperature of hominins is equal to (or lower than) the current body temperature of humans, it is evident that the minimal number of feeding hours becomes “large,” i.e., greater than 8 h. For example, upon considering Δ*T* = 0 and $N=N_{\text{sapiens}}$, the rather high value of $H_{\text{min}}\approx 9.3$ is obtained. On the other hand, for Δ*T* = +2°C and $N=N_{\text{sapiens}}$, one arrives at $H_{\text{min}}\approx 7.4$, which is more reasonable.

**Figure 6:**
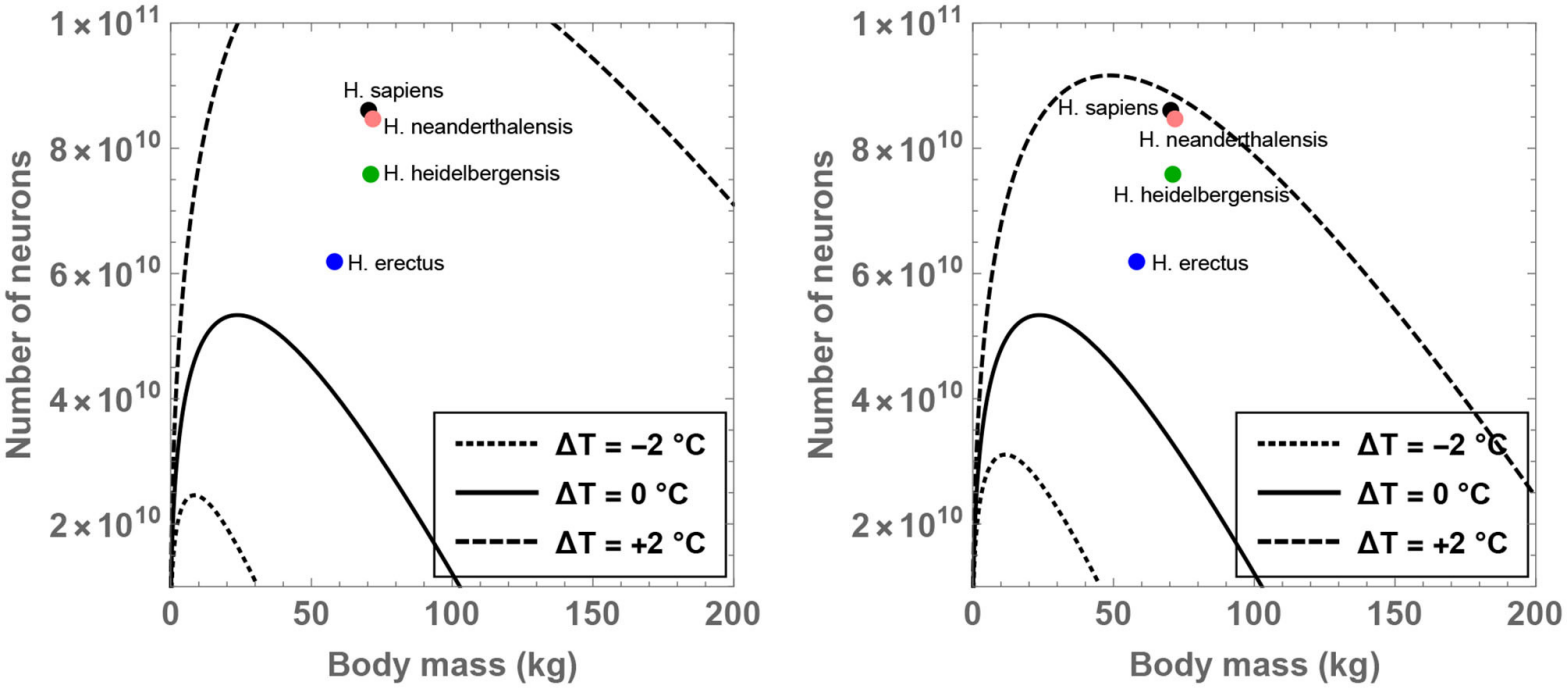
Compatible combinations of the number of neurons (N) and body mass (*M*_BD_) are shown for putative primates that engage in 8 hours of feeding per day; regions below the curves represent the permitted values. The residual temperature (Δ*T*) quantifies the deviation of body temperature from the conventional value of 37°C. The left and right panels were derived using activation energies of 0.65 and 0.94 eV, respectively. The black, orange, green, and blue dots show the values of N and *M*_BD_ for *H. sapiens, H. neanderthalensis, H. heidelbergensis*, and *H. erectus*, respectively.

Lastly, it is worth relaxing a prominent assumption of Section 2 and exploring the ensuing consequences. Hitherto, the “canonical” activation energy of E≈0.65 eV (Brown et al., 2004) was implicitly utilized, but it has been suggested that adopting E≈0.94 eV might be more accurate for moderately-sized mammals (Downs et al., 2008). Upon recalculating N as a function of *M*_BD_ for this activation energy, one can evaluate the analog of Figure 1. The results are plotted in Figure 6, from which it is apparent that a higher activation energy brings about smaller upward or downward shifts in N for a given value of Δ*T*.

## 4. Discussion and Conclusions

In this work, the potential role of body temperature in governing the tradeoff between body size and number of neurons (i.e., brain size) arising from metabolic constraints was explored by means of a heuristic theoretical model. The ensuing ramifications must be accordingly interpreted with the appropriate degree of caution due to the inherent uncertainties and assumptions in the model.

There are two major qualitative outcomes that appear to be generally valid *prima facie*. First, even modest (i.e., a couple of °C) changes in the average body temperature across a species may engender substantial revisions of the permitted brain and body sizes. Second, it was determined that elevating the body temperature could drive putative hominins toward higher number of neurons, increased brain-to-body mass ratio, and fewer hours spent on foraging to cover metabolic needs. The converse effects are ostensibly true when body temperature is decreased. From a quantitative standpoint, it was estimated that a body temperature approximately 1–2°C higher (at the minimum) than *T*_0_≈37°C might suffice to offset metabolic costs and thence permit the evolution of hominins such as *H. erectus* and subsequent species; on the other hand, *ceteris paribus*, the canonical human body temperature would pose difficulties for the advent of these species.

It is necessary, however, to recognize that a higher body temperature could entail certain costs that serve to diminish its value vis-à-vis permitting larger number of neurons. For instance, maintaining a higher body temperature, especially in colder periods or regions, is anticipated to be more challenging because the heat dissipated is roughly proportional to the temperature difference between the body temperature and the environment (Bergman et al., 2020); sustaining a higher body temperature is therefore harder because the heat loss would be commensurately higher. Other consequences of increasing the body temperature (both positive and negative) in connection with thermoregulation, foraging, fecundity, locomotion, and life cycles of organisms are elucidated in Angilletta (2009), Clarke (2017), and Garland et al. (2022); the details are not explicitly spelled out since the ramifications are subtle and/or equivocal in some cases.

The next question that springs to the forefront is whether a higher body temperature would have been feasible in the past. A recent study asserted that the body temperature of humans has decreased (in a linear fashion) by 0.6°C in the past ~150 years (Protsiv et al., 2020). In a similar vein, the data analysis conducted by Gurven et al. (2020) indicates that temperature has declined rapidly in recent times, at the rate of ~0.05°C/year. However, these publications must be interpreted with due caution because of the accompanying changes in how the temperatures were taken and recorded over time. Bearing this important caveat in mind, for the sake of argument, let us suppose that the conclusions from the aforementioned papers are realistic.

The fact that this marked change in the temperature purportedly occurred over a relatively short timescale lends some credence to the notion that hominins may have possessed higher or lower body temperatures when benchmarked against present-day humans. Note that the emergence of relatively large-brained hominins, loosely commencing with *H. erectus*, transpired during the interval of ~0.2–2 Mya (Tattersall, 2012; Coolidge and Wynn, 2018; Reich, 2018; Lingam and Loeb, 2021). Hence, the requisite average rate of temperature variation is ~10^−5^ to ~10^−6^°C per year under the supposition of linear scaling to achieve a cumulative increase of the body temperature by Δ*T* = 2°C over the chosen timescale. In turn, the linear model translates to a fairly minimal increment of ~10^−3^−10^−4^°C per century—whether this trend is sustainable over a long timescale is certainly debatable, but it ought not be altogether dismissed *a priori*.

One can also ask the converse question of what would happen if lower body temperatures were prevalent in hominins. Before addressing this issue, it is worth examining the thermal data from primates. The body temperature of the western lowland gorilla (*G. g. gorilla*) is around 35.5°C (Brown and Finnegan, 2007), which is approximately 1.5°C lower than the fiducial value employed in our treatment. Several species of *Euarchonta* (which encompasses primates) evince body temperatures <35 °C (Clarke et al., 2010). On the other hand, both chimpanzees (*P. troglodytes*) and Bornean orangutans (*P. pygmaeus*) have body temperatures nearly equal to that of humans (Just et al., 2019). Thus, perhaps the most parsimonious hypothesis is that the mean body temperature has not undergone alteration during the course of hominin evolution. In this scenario, the effects of body temperature may prove to be minimal and other factors would duly need to be exclusively considered to explain the shifts in brain and body sizes of hominins over time^1^.

With that said, the possibility that hominins had lower or higher body temperatures cannot be definitively ruled out, given that dietary restrictions are documented to play a vital role in regulating human body temperature (Lane et al., 1996). A panoply of other processes, both endogenous and exogenous, could trigger changes in body temperature, owing to which ascertaining the latter's trajectory in hominins is far from straightforward. For instance, body temperature is modulated to varying degrees by fluctuations in environmental temperature, energetic costs and benefits associated with thermoregulation, alterations of life cycles, and the extent of predation and competition for resources, to name a handful (Angilletta, 2009). As an interesting aside, it is worth recognizing that the climate of East Africa apparently witnessed substantive fluctuations in the Quaternary (Potts, 2013; Antón et al., 2014; Maslin et al., 2014), which might have affected not just hominin evolution broadly speaking but the brain sizes as well (Shultz and Maslin, 2013).

In the event that Δ*T* < 0, i.e., hominins had lower body temperatures, the metabolic tradeoffs are rendered far more stringent. In heuristic terms, as per the results presented earlier, one may expect fewer number of neurons, decreased brain-to-body mass ratio and more hours spent on foraging to cover metabolic needs. In other words, the necessity for a counteracting mechanism is amplified commensurately. An evident solution is to enhance the energetic intake per hour of feeding. This outcome can be achieved by changes in diet and foraging (Milton, 1999; Doḿınguez-Rodrigo et al., 2005; Zink and Lieberman, 2016), or through the consumption of cooked foods (Carmody and Wrangham, 2009; Carmody et al., 2011). Thus, in case putative hominins were characterized by lower body temperatures, the “cooked food” hypothesis pioneered, *inter alia*, by Richard Wrangham and collaborators (Wrangham et al., 1999; Wrangham, 2009, 2017) could acquire greater significance. While an unequivocal picture of whether genus *Homo* was capable of fire control in the early and mid Pleistocene, is missing heretofore (Sandgathe and Berna, 2017), recent developments on this front—especially in connection with the site FxJj20 in Koobi Fora, Kenya (Hlubik et al., 2017, 2019)—seem promising (Gowlett, 2016; Wrangham, 2017).

To sum up, if the body temperature of hominins was higher or lower than current humans by a couple of °C, the analysis herein indicates that this shift may have potentially facilitated perceptible changes in their body sizes and number of neurons insofar as metabolic constraints are concerned. Therefore, at the minimum, there are two interconnected areas that merit further investigation: (i) accurately gauging hominin body temperature by synthesizing empirical and theoretical studies in physiology, anthropology, paleontology, and genomics, and (ii) deploying the inferred temperature in sophisticated thermal models (e.g., with quadratic corrections) to comprehend how metabolic (*E*_BD_ and *E*_BR_) and feeding (*E*_IN_) costs along with the resultant brain and body sizes are modulated. Needless to say, aside from the possible role of temperature, a multitude of extrinsic and intrinsic factors such as the foraging efficiency, cumulative culture, diet, and climate must be taken into account.

## Data Availability Statement

The original contributions presented in the study are included in the article/supplementary material. Further inquiries regarding the modeling and/or data can be directed to the corresponding author/s.

## Author Contributions

The author confirms being the sole contributor of this work and has approved it for publication.

## Author Disclaimer

The author is solely responsible for any potential errors and oversights that are present in this work.

## Conflict of Interest

The author declares that the research was conducted in the absence of any commercial or financial relationships that could be construed as a potential conflict of interest.

## Publisher's Note

All claims expressed in this article are solely those of the authors and do not necessarily represent those of their affiliated organizations, or those of the publisher, the editors and the reviewers. Any product that may be evaluated in this article, or claim that may be made by its manufacturer, is not guaranteed or endorsed by the publisher.

## Footnotes

^1^The caveat at the end of Section 1 should therefore be borne in mind.
